# Fecal microbiota transplantation: a novel strategy and challenges in the adjuvant treatment of bladder Cancer

**DOI:** 10.3389/fmicb.2026.1756107

**Published:** 2026-02-03

**Authors:** Xinwei Liu, Zhiqiang Chen, Yichen Lu, Yifan Wu, Yongneng Huang, Yuwei Zhang, Menglu Li, Ninghan Feng

**Affiliations:** 1 Department of Urology, Jiangnan University Medical Center, Wuxi, China; 2 Institute of Urology, Wuxi School of Medicine, Jiangnan University, Wuxi, China; 3 Department of Urology, Wuxi No. 2 People's Hospital, Nanjing Medical University, Nanjing, China; 4 Department of Urology, Wuxi Medical Center, Nantong University, Nantong, China

**Keywords:** adjuvant therapy, bladder cancer, fecal microbiota transplantation, gut microbiota, gut-bladder axis

## Abstract

The clinical management of bladder cancer faces major challenges due to treatment resistance and recurrence, which require the development of new adjuvant strategies. The role of the gut microbiome in influencing bladder cancer progression and treatment response through the “gut-bladder axis” is gaining recognition. This understanding provides a theoretical rationale for exploring microbiota-targeting interventions, such as fecal microbiota transplantation (FMT). As a method capable of thoroughly reshaping the gut microbiota, FMT may have broad clinical potential. This review systematically explores the possible role of FMT in treating bladder cancer. It begins by summarizing the observational and causal evidence linking gut microbiota dysbiosis to bladder cancer, which forms the rationale for considering FMT as an intervention. Then, it discusses how FMT might improve therapeutic effectiveness, including regulation of microbial metabolites (such as short-chain fatty acids, tryptophan, and bile acids), repair of the intestinal barrier, induction of epigenetic reprogramming and modulation of the urinary microbiota. The review also considers potential scenarios for combining FMT with existing adjuvant therapies, including immunotherapy, chemotherapy, and radiotherapy. Finally, it objectively evaluates the key challenges in translating FMT into clinical practice, including effectiveness, safety, standardization, and regulatory or ethical issues, and outlines future directions. By synthesizing current evidence, this review highlights FMT as a potentially promising and innovative adjuvant strategy worthy of further investigation, which, if validated, could contribute to overcoming current therapeutic challenges in bladder cancer.

## 1 Introduction

Bladder cancer stands as one of the most commonly diagnosed malignancies globally, with approximately 614,000 new cases and 220,000 deaths in 2022, highlighting its significant incidence and mortality (Bray et al., 2024). This disease imposes a substantial psychological and financial burden, driven by high care costs from frequent recurrence, treatment complications, and diminished quality of life (Lobo et al., 2022; Joyce et al., 2023; Francolini et al., 2024; Scilipoti et al., 2025). A central challenge in the clinical management of bladder cancer is the high rate of recurrence and the development of resistance to various treatments. Many patients with muscle-invasive bladder cancer (MIBC) experience recurrence or metastasis after radical cystectomy (Compérat et al., 2022; Lopez-Beltran et al., 2024). Although immune checkpoint inhibitors and novel chemotherapy regimens show promise, their limited overall efficacy leaves most patients facing drug resistance (Lopez-Beltran et al., 2024; Dyrskjøt et al., 2023). These persistent challenges underscore the urgent need for innovative adjuvant strategies to enhance therapeutic efficacy and overcome treatment resistance.

Over recent decades, research has progressively unveiled the profound influence of the gut microbiome on host physiology, spanning metabolism, immune regulation, and even behavioral processes (Pepke et al., 2024; Sen et al., 2021; Shim et al., 2023; Fan and Pedersen, 2021; Manor et al., 2020). Its significant role in tumor development, progression, and treatment response is increasingly recognized across diverse malignancies, including lung, breast, colorectal, and prostate cancers, as well as melanoma (Wong and Yu, 2023; Pernigoni et al., 2021; Jin et al., 2019; Gopalakrishnan et al., 2018; Schettini et al., 2024). Accumulating evidence further implicates the gut microbiome in bladder cancer pathogenesis and treatment outcomes, supporting the concept of a bidirectional “Gut-Bladder Axis” (Roje et al., 2024; Wang et al., 2024; Fidelle et al., 2023). This axis is hypothesized to modulate the bladder tumor microenvironment through several key mechanisms. These include immune regulation, microbial metabolite secretion, and circulating non-coding RNA networks, thereby ultimately influencing cancer progression and therapeutic response (Bredon et al., 2024; Zhu et al., 2020; Wu et al., 2025; Xu et al., 2024). Collectively, these findings offer crucial insights into the mechanisms that drive bladder cancer recurrence, progression, and treatment resistance.

Consequently, targeting the gut microbiota is regarded as a promising and exploratory strategy to address current clinical challenges in bladder cancer. Among various microbiome-targeting interventions, FMT is often highlighted for its considerable potential in oncology. Its ability to rapidly and comprehensively reshape the gut microbial ecosystem is thought to be a feature that could hold particular promise for enhancing the efficacy of immunotherapy (Kim et al., 2024; Wang et al., 2024; Huang et al., 2022; Bohm et al., 2025). This review aims to systematically explore the potential value of FMT in the treatment of bladder cancer. First, we will delineate the theoretical foundation for its application. Next, we will elaborate on the potential mechanisms through which FMT influences therapeutic efficacy via the “gut-bladder axis.” Finally, we will objectively discuss the key challenges and future prospects for its clinical translation.

## 2 Targeting the gut microbiota: the theoretical basis and evidence for FMT in bladder cancer

Although prospective studies directly comparing gut microbiota composition between healthy individuals and bladder cancer patients remain limited, existing evidence supports a strong association between gut dysbiosis and this cancer (Table 1). Consistently observed in patients are reduced gut alpha diversity and decreased abundance of genera such as *Prevotella*, *Bacteroides*, and *Clostridium* (He et al., 2020; Qin et al., 2022). Interestingly, these same genera are frequently enriched in patient urine or tissues, as demonstrated by studies profiling urinary microbiota (Zeng et al., 2020; Oresta et al., 2021; Parra-Grande et al., 2021). Given that the gut is considered a key source of urinary microbes, these parallel findings could suggest translocation from the gut to the bladder. If such translocation occurs, these bacteria might then influence bladder cell function through metabolite-host interaction (Magruder et al., 2019).

**Table 1:** Bladder cancer-associated gut microbiota alterations: A summary of observational evidence.

Study: year, First author	Race	Specimen	Number of participants	Microbiome assessment method	Main changing microbiota
He et al. (2020)	Human	Stool	NC:16 BC:26	Pyrosequencing and qPCR	BC group: reduced *Clostridium cluster XI* and *Prevotella*
Qin et al. (2022)	Human	Stool	NC:15 BC:32	Metagenomic Sequencing	BC group: decreased relative abundance of 19 taxa (incl. *Clostridium* & *Prevotella*)
Bukavina et al. (2022)	Human	Stool	NC:32 BC:29	ITS Sequencing	BC group: increased fungal *α*-diversity and enriched *Dothideales*, *Hypocreales*, *Tremellales*
Chorbińska et al. (2023)	Human	Stool	NC:7 BC:23	16S rRNA gene V3–V4 regions	No significant differences in *α*/*β*-diversity or differentially abundant taxa between bladder cancer patients and controls
Li et al. (2025)	Human	Stool	NC:22 BC:50	16S rRNA gene V3–V4 regions	BC group: decreased α-diversity and depletion of Bacteroides, Prevotella, & Parabacteroides relative to the NC group

This table summarizes observational studies comparing gut microbiota between bladder cancer (BC) patients and non-cancer (NC) controls. A notable trend across several studies is the consistent reduction of specific bacterial genera, such as Clostridium and Prevotella, in BC patients. However, findings on alpha-diversity are inconsistent, with reports of either decrease, increase, or no significant change. The most recent studies further suggest a possible shift in phylum-level composition (e.g., decreased Firmicutes, increased Fusobacteria) and a depletion of Bacteroides. These alterations point to a potential dysbiotic gut microenvironment that may be linked to bladder cancer, though heterogeneity in study design and microbiome assessment methods should be considered when interpreting these associations. HC: Healthy Control; BC: Bladder Cancer; qPCR: Quantitative Polymerase Chain Reaction; ITS: Internal Transcribed Spacer.

However, these taxonomic differences cannot establish causality. To address this limitation, Mendelian randomization (MR) analyses have been employed (Table 2). Synthesis of current MR evidence reveals that genetically predicted abundance of specific gut taxa, such as the *Eubacterium coprostanoligenes* group, is significantly and causally associated with increased bladder cancer risk (Che et al., 2025; Zhang et al., 2023). This finding aligns with prior observational data, which provides genetic support for the premise that gut dysbiosis may play a role in bladder carcinogenesis. Nonetheless, these MR-derived conclusions require cautious interpretation, as their validity is dependent on the strength of the genetic instruments and can be susceptible to pleiotropic bias (Burgess and Thompson, 2011). This need for caution is further underscored by observed variations and inconsistencies within the MR evidence itself. For instance, while *Bifidobacterium* is commonly considered a probiotic genus, one MR study identified it as a risk factor for bladder cancer. Furthermore, effects can diverge even at the species level within a single genus, as illustrated by the opposing roles of *Bacteroides salyersiae* (risk factor) and *Bacteroides dorei* (protective factor) reported in the same analysis. These apparent discrepancies may be partly attributable to methodological heterogeneity across studies. A closer examination reveals variations at several levels: the genome-wide significance thresholds for selecting instrumental variables (from *p* < 5 × 10^−8^ to *p* < 1 × 10^−5^), parameters for linkage-disequilibrium clumping (r^2^ from <0.001 to <0.01; distances from 500 kb to 10,000 kb), and inconsistent emphasis on excluding weak instruments (e.g., F-statistic > 10). Additionally, issues of sample overlap and population heterogeneity merit careful consideration. Several key studies (e.g., 117, 118, 121) derive their instrumental variables from the same large-scale, multi-ethnic microbiome GWAS (the MiBioGen consortium, *n* = 18,340). While this shared data source provides a valuable foundation, it also introduces the potential for correlated biases if the outcome GWAS samples are not fully independent. Moreover, although the MiBioGen cohort includes participants of European, African, and Asian ancestry, the predominance of European individuals (≈72%) means that the genetic instruments are optimized for, and their performance may be most reliable in, populations of European descent. The generalizability of these causal estimates to other ancestral groups remains uncertain. When compounded with inherent challenges such as limited microbial taxonomic resolution in GWAS and collinearity among taxa, these methodological and population-level differences collectively constrain the definitiveness of causal inferences and underscore the probabilistic nature of current MR findings. Therefore, current MR evidence is best interpreted as highlighting probabilistic causal targets rather than delivering definitive conclusions. Future research must prioritize functional validation through refined experimental designs. The ultimate goal is to definitively elucidate how these microbes contribute to pathogenesis, thereby providing a robust foundation for microecological interventions.

**Table 2:** Mendelian randomization study: causal effects of specific gut microbiota on bladder cancer risk.

Study: year, First author	Exposure (microbial classification)	OR	95% CI	*P*	Association
Mingdong et al. (2023)	*Genus Bifidobacterium*	1.496	(1.04, 2.15)	0.03	Risk factor
Yin et al. (2023)	*Genus Allisonella*	0.55	(0.37, 0.82)	0.00337	Protective factor
	*Eubacterium coprostanoligenes*	4.27	(1.56, 11.68)	0.00472	Risk factor
Zhang et al. (2024)	*Eubacterium ruminantium group*	1.33	(1.09, 1.61)	0.004	Risk factor
	*Ruminococcaceae UCG013*	1.64	(1.17, 2.31)	0.004	Risk factor
	*Ruminococcaceae UCG004*	0.73	(0.56, 0.97)	0.027	Protective factor
	*Eubacterium brachy group*	0.80	(0.66, 0.97)	0.026	Protective factor
Wei et al. (2025)	*Dorea*	2.20	(1.29, 3.75)	0.0037	Risk factor
Che et al. (2025)	*Genus Streptococcus*	1.26	(1.03, 1.55)	0.025	Risk factor
	*Species Bacteroides salyersiae*	1.13	(1.01, 1.26)	0.036	Risk factor
	*Species Bacteroides dorei*	0.65	(0.53, 0.80)	<0.001	Protective factor
Yang et al. (2025)	*Order Enterobacteriales*	0.53	(0.37, 0.74)	<0.001	Protective factor
	*Family Enterobacteriaceae*	0.53	(0.37, 0.74)	<0.001	Protective factor
	*Genus Bilophila*	1.38	(1.05, 1.80)	0.01905	Risk factor
	*Genus Oscillibacter*	0.77	(0.63, 0.94)	0.00885	Protective factor
	*RuminococcaceaeNK4A214* *group*	0.75	(0.58, 0.97)	0.02860	Protective factor

This table selectively presents key causal evidence from Mendelian randomization studies linking specific gut microbiota features to bladder cancer risk. It highlights consistent patterns: genera such as Streptococcus, Dorea, and Bilophila are identified as risk factors, while features within the Enterobacteriales order and genus Oscillibacter appear protective. A notable finding is the species-specific effect within the same genus, exemplified by *Bacteroides salyersiae* (risk) versus *Bacteroides dorei* (protective). These genetically supported causal associations point to specific microbial taxa that warrant further investigation as potential mechanistic or therapeutic targets in bladder cancer. OR: Odds Ratio; CI: Confidence Interval.

Despite methodological limitations, evidence from both observational and genetic studies collectively supports targeting the gut microbiota in bladder cancer. FMT offers a potential strategy to reverse such dysbiosis by introducing a complete and healthy microbial ecosystem (Yadegar et al., 2024). Its application is premised on the hypothesis that restoring a healthy gut microbiota could counteract identified procarcinogenic effects or potentiate the efficacy of existing adjuvant therapies, such as immune checkpoint inhibitors or chemotherapy. Future studies should integrate more sophisticated experimental designs and functional validation to elucidate the specific mechanisms by which gut microbes influence bladder cancer progression. In this process, FMT can serve a dual role, functioning both as a therapeutic modality and as a platform for mechanistic inquiry, thereby laying the groundwork for developing microecology-based precision therapies.

## 3 Potential mechanisms of FMT in bladder cancer treatment through multiple pathways

### 3.1 Metabolite modulation: FMT as a delivery platform for bioactive metabolites

#### 3.1.1 Tryptophan

Tryptophan metabolism as a gut microbiota-regulated pathway offers crucial mechanistic framework for understanding how FMT might influences bladder cancer. This catabolic process occurs through three major routes: the kynurenine pathway, serotonin pathway, and microbial indole pathway, with their balance critically shaping the tumor immune microenvironment (Liu and Zhai, 2021).

Recent transcriptomic analyses reveal that the overall expression levels of rate-limiting enzymes in tryptophan metabolism, including IDO1, KMO, and TPH1, are significantly downregulated in bladder tumor tissues compared to normal tissues (Lu et al., 2025). Nevertheless, this overall trend may obscure critical biological behaviors within specific cellular populations or tumor subtypes. IDO and TDO are key enzymes that catalyze the rate-limiting step of the kynurenine pathway, generating N-formylkynurenine. Typically expressed in tumor cells and myeloid cells, such as antigen-presenting cells, these enzymes serve as core molecules mediating the formation of an immunosuppressive microenvironment. The underlying mechanism involves IDO-mediated depletion of local tryptophan and accumulation of metabolites, including kynurenine, which directly suppresses T cell proliferation while enhancing the function of regulatory T cells (Treg), thereby facilitating immune escape (Liu and Zhai, 2021; Le Floc'h et al., 2011). Previous studies have predominantly focused on the functional role of IDO1, revealing its elevated expression in urothelial carcinoma compared to normal tissues, with differential expression patterns between non-muscle-invasive bladder cancer (NMIBC) and muscle-invasive bladder cancer (MIBC). Functionally, silencing IDO1 has been demonstrated to significantly suppress bladder tumor cell invasion, proliferation, and the epithelial-mesenchymal transition process (Zhang et al., 2019; Santos et al., 2022). This apparent paradox between overall downregulation and specific functional criticality suggests that IDO1 expression may exhibit significant intertumoral and intratumoral heterogeneity. Its pro-tumorigenic functions might not depend on the average expression level across bulk tumor tissue, but rather be driven by localized high expression in specific cellular subsets within the tumor microenvironment, such as myeloid cells. Given its potent immunosuppressive function, IDO1 remains a crucial therapeutic target. This is underscored by the significantly elevated kynurenine-to-tryptophan ratio in patient plasma and urine, which confirms the activation of this pathway in bladder cancer and explains its key role in shaping an immunosuppressive tumor microenvironment (Lee et al., 2021).

Conversely, the serotonin pathway has been found to promote tumor migration, while its antagonists can suppress the growth of bladder tumor cells (Siddiqui et al., 2006). In contrast, the microbial indole pathway demonstrates a clear protective role. Recent research has revealed a deficiency of indole-producing bacteria in bladder cancer patients. Experimentally, supplementing these bacteria elevates systemic indole-3-acetic acid (IAA) levels. This increased IAA enhances ferroptosis sensitivity in bladder cancer cells via the AhR-FASN axis and then suppresses tumor progression (Li et al., 2025).

In this context, the proposed core value of FMT lies in its potential to transcend the limitations of single molecules and systematically remodel the overall network balance of tryptophan metabolism. Theoretically, by introducing a functionally intact microbial community, FMT could exert multi-faceted effects: modulating the immunosuppressive kynurenine pathway, suppressing pro-tumorigenic serotonin signals, and restoring the protective microbial indole pathway (Huang et al., 2022; Wang et al., 2025). Therefore, multi-targeted and holistic modulation of tryptophan metabolism through FMT may represent a potential mechanism for reversing the immunosuppressive microenvironment in bladder cancer and enhancing treatment sensitivity. It is important to emphasize, however, that while the individual roles of these tryptophan-derived pathways in bladder cancer are increasingly clear, the synthesized hypothesis that FMT can therapeutically coordinate a rebalance among them remains to be directly validated in the context of bladder cancer.

#### 3.1.2 Short-chain fatty acids

Short-chain fatty acids (SCFAs) are hypothesized to be key mediators of the putative therapeutic effects of FMT. By transferring SCFA-producing bacteria from healthy donors, FMT is postulated to elevate systemic levels of metabolites such as acetate, propionate, and butyrate, which could correct the SCFA imbalance observed in bladder cancer patients in theory (He et al., 2020). Among various SCFAs, butyrate stands out as the most biologically active. Through mechanisms such as inhibiting histone deacetylase (HDAC) and activating G-protein coupled receptors (GPR41/GPR43), it not only directly induces cell cycle arrest and apoptosis in bladder cancer cells, but also systemically enhances the cytotoxic function of CD8^+^ T cells and modulates inflammatory responses (Yang et al., 2020; Luu et al., 2021; Luu et al., 2018). These systemic immunomodulatory effects complement the direct inhibitory impact of butyrate on bladder cancer cells. Experimental studies have demonstrated that butyrate and butyrate-producing bacteria can induce cell cycle arrest, upregulate pro-apoptotic proteins, and suppress migratory capacity in bladder cancer cells (Wang et al., 2021; Wang et al., 2020). Meanwhile, acetate and propionate contribute essential immunomodulatory functions. For instance, propionate suppresses pro-tumorigenic Th2 responses and M2 macrophage polarization, whereas acetate alleviates macrophage-driven inflammation by inhibiting HIF-*α*-dependent glycolysis (Hays et al., 2024; Li et al., 2024; Duan et al., 2023). Taken together, FMT represents a holistic strategy theorized to remodel the SCFA profile and restore an antitumor immune microenvironment. However, this therapeutic hypothesis, while grounded in strong experimental evidence for SCFA functions, requires direct validation in clinical studies to confirm that FMT can systemically deliver a protective SCFA profile in bladder cancer patients.

#### 3.1.3 Bile acids

Bile acids (BAs) are proposed to be another key pathway through which FMT could modulate bladder cancer progression. By transferring a complete set of microbial bile-acid-transforming enzymes, FMT could correct the host’s dysregulated bile acid metabolism (Allegretti et al., 2020; Zhang et al., 2025). Furthermore, gut microbiota primarily shapes the bile acid profile through expression of key enzymes like bile salt hydrolase (BSH), thereby generating diverse bile acid species that finely regulate downstream signaling pathways centered on the farnesoid X receptor (FXR) (Tong and Lou, 2025; Cai et al., 2022). Upon binding to bile acids, FXR forms a heterodimer with the retinoid X receptor (RXR) and translocates into the nucleus to regulate gene transcription (Tong and Lou, 2025). This receptor demonstrates higher expression in normal bladder tissues compared to tumor tissues and has been shown to suppress malignant behaviors (Lai et al., 2022). Furthermore, bladder cancer exhibits additional dysregulation in bile acid signaling. For instance, levels of oncogenic hydrophobic bile acids such as lithocholic acid are significantly elevated, suggesting that dysregulated bile acid metabolism may contribute to disease progression (Režen et al., 2022; Tan et al., 2017). The core potential of FMT lies in its ability to reshape this aberrant metabolic landscape. For example, introducing functional bacteria like *Akkermansia* can promote the production of the protective bile acid UDCA, thereby activating FXR and suppressing pro-carcinogenic pathways such as NF-κB (Bai et al., 2025; Wu et al., 2021). Consequently, FMT holds the theoretical potential to reprogram this cascade and shift the host’s metabolic state toward tumor suppression. It is crucial to note, however, that this potential remains hypothetical. Its realization depends on future validation demonstrating that FMT can durably establish a functional microbial network capable of producing a tumor-suppressive bile acid profile in patients.

### 3.2 FMT for barrier repair and inflammation control

In bladder cancer patients, prevalent gut dysbiosis and impaired intestinal barrier function are considered to constitute the pathological basis of a disrupted gut-bladder axis (He et al., 2020; Qin et al., 2022). This disruption of intestinal barrier integrity, often termed “leaky gut,” allows bacterial lipopolysaccharide and other pathogen-associated molecular patterns to translocate into systemic circulation. This process triggers chronic, low-grade systemic inflammation, which may potentially accelerate bladder cancer progression (Wang et al., 2022). FMT is proposed as a direct approach to rectifying this defect by introducing a complete, healthy microbial community. A key proposed mechanism involves restoring beneficial bacteria that produce short-chain fatty acids, particularly butyrate. As a crucial energy source for colonic epithelial cells, butyrate significantly upregulates the expression of tight junction proteins such as ZO-1 and Occludin, thereby directly strengthening and repairing the intestinal physical barrier (Hays et al., 2024; He et al., 2018). Simultaneously, the reconstructed microbial ecology could help maintain gut immune homeostasis, mitigating the persistent damage to the barrier caused by local inflammation. As intestinal barrier function is restored, the levels of systemic pro-inflammatory cytokines (e.g., IL-1β, IL-6) are effectively reduced (He et al., 2018). These cytokines are well-established tumor promoters that activate key signaling pathways, such as NF-κB and AP-1, thereby driving tumor cell proliferation, invasion, and anti-apoptosis (Xia et al., 2018). Given that chronic systemic inflammation is closely linked to local urinary tract inflammation, which plays a critical role in bladder cancer pathogenesis, FMT could potentially hold promise for curbing the pro-carcinogenic inflammatory environment at its source. Theoretically, by serving as a “firewall” and “anti-inflammatory agent,” it may indirectly suppress inflammation-driven malignant progression in the bladder. However, while the “leaky gut” model and its link to systemic inflammation provide a compelling mechanistic rationale, the hypothesis that FMT can suppress bladder cancer progression by specifically restoring intestinal barrier integrity awaits direct experimental and clinical validation in the context of this disease.

### 3.3 Epigenetic reprogramming: FMT as a remote regulator for gene expression

Epigenetic modifications, specifically disruptions in DNA methylation and regulation by non-coding RNAs, are key drivers of malignant progression and chemoresistance in bladder cancer (Li et al., 2025). By remodeling the gut microbiota, FMT could remotely and systemically intervene in these two parallel mechanisms, thereby reprogramming the host’s gene expression profile (Zhang et al., 2025). At the DNA methylation level, bladder cancer is characterized by the hypermethylation-induced silencing of tumor suppressor genes and the hypomethylation-driven activation of oncogenes. These alterations are closely associated with tumor proliferation, invasion, and cisplatin resistance (Li et al., 2025; Maesaka et al., 2022; Ma et al., 2024; Li et al., 2023). The microbiota introduced by FMT metabolically generate key molecules such as short-chain fatty acids, folate, and S-adenosylmethionine. These compounds can act as methyl donors or directly modulate DNA methyltransferase activity, thereby systemically correcting aberrant DNA methylation patterns. Clinical studies have observed the remodeling of the host DNA methylome by FMT in other diseases, providing a proof-of-principle for its potential application in bladder cancer (Zhang et al., 2025; Stols-Gonçalves et al., 2023; Zhang et al., 2023). At the non-coding RNA level, various miRNAs such as miR-320a-3p, long non-coding RNAs such as LINC00665, and circular RNAs such as Hsa_circ_0001583, are dysregulated in bladder cancer and mediate key malignant behaviors (Liu et al., 2024; Li et al., 2023; Lv et al., 2024; Zou et al., 2024). For instance, the downregulation of miR-320a-3p leads to overexpression of its target IGF2BP3, which in turn stabilizes HMGB1 mRNA and promotes tumor progression through the IGF2BP3-HMGB1 axis (Lv et al., 2024). Similarly, the long non-coding RNA DLX6-AS1 is upregulated and functions as a competing endogenous RNA (ceRNA)for miR-195-5p, thereby activating the VEGFA/Ras/Raf/MEK/ERK signaling cascade to drive proliferation and metastasis (Wang et al., 2020). Another example is LINC00858, which promotes invasion by sequestering miR-3064-5p and upregulating CTGF (cellular communication network factor 2) (Huang et al., 2021). Taken together, given that FMT may indirectly modulate the expression of such non-coding RNAs by altering specific gut microbial populations, it is hypothesized to remotely reprogram the epigenetic landscape of bladder cancer through these coordinated mechanisms, thereby reshaping downstream target gene networks and influencing cancer progression (Zhu et al., 2020; Ma et al., 2025). It is critical to distinguish, however, between the well-established role of epigenetic dysregulation in bladder cancer and the proposed ability of FMT to reverse it. While preclinical evidence and parallels from other diseases are supportive, direct clinical evidence validating FMT’s epigenetic efficacy in bladder cancer is awaited.

### 3.4 Bladder microenvironment reshaping: FMT as an indirect modulator of urinary flora

Beyond the systemic and epigenetic influences outlined earlier, the gut bladder axis may also contribute to shaping the local tumor microenvironment of the bladder by modulating the urinary microbiota. Once considered largely sterile, the urinary tract is now known to host a unique microbial community, and its composition can interact with both the gut microbiota and the host immune system (Stone, 2023). Multiple studies have revealed significant differences in the urinary microbiota between bladder cancer patients and healthy individuals. For example, *Veillonella* has been found to be enriched in the urine of bladder cancer patients in several studies, and it appears particularly prominent in patients with recurrence after surgery for both muscle invasive and non- muscle-invasive bladder cancer, suggesting a potential link to disease progression and recurrence (Hrbáček et al., 2023; Hussein et al., 2023; Hussein et al., 2021). Importantly, a case report of *Veillonella* bacteremia in a bladder cancer patient provides supporting evidence that this bacterium may originate from the gut and spread through the bloodstream to various sites, including the urinary system (Cobo et al., 2020). Furthermore, other studies have identified additional bacterial genera that are relatively enriched in the urine of bladder cancer patients, such as *Fusobacterium*, *Achromobacter*, and *Brucella* (Hussein et al., 2021; Bučević Popović et al., 2018). While the specific mechanisms through which the urinary microbiota influences the development and progression of bladder cancer are not yet fully understood, existing evidence strongly underscores its significance as a valuable area of research.

It is important to note that the gut is likely a major source of microorganisms for the urinary tract (Magruder et al., 2019). In addition to hematogenous spread, the anatomical proximity of the anus and urethra suggests possible ascending colonization pathways, enabling certain gut bacteria to establish residence in the urogenital tract (Curtis et al., 2025). Concurrently, metabolites produced by the gut microbiota can influence the colonization landscape of the urogenital tract by modulating systemic immune status or altering urinary physicochemical properties, such as pH (Colella et al., 2023). Dysbiosis of the urinary microbiota may further disrupt the local bladder microenvironment. Current perspectives propose that uropathogenic microbes may participate in bladder carcinogenesis through multiple pathways, including cellular microenvironment modulation by the microbiome, bacterial-induced inflammation, direct DNA damage caused by bacterial toxins, and modulation of intracellular signaling pathways that exert either pro tumor or anti-tumor effects (Butt and De Biase, 2025). For instance, the bladder urothelium is coated with glycosaminoglycans, components of the extracellular matrix, which help separate it from urine. Some microorganisms can secrete matrix metalloproteinases (MMPs), collagenases, and other enzymes that degrade the structure of glycosaminoglycans, leading to abnormal breakdown or remodeling of the extracellular matrix. This promotes bacterial dissemination and compromises the epithelial barrier (Alfano et al., 2016; De Gregorio et al., 2022). Simultaneously, this process may trigger an inflammatory response, which can, in turn, interfere with intracellular signaling pathways, particularly the STAT3 pathway. This pathway has been shown to play a key role in the initiation and proliferation of bladder cancer (Sadhukhan et al., 2024). Therefore, FMT, as an approach to reshape the gut microbiota, may indirectly influence the urinary microbiota and subsequently affect the progression of bladder cancer. Although evidence in this field is still accumulating, the urinary microbiota is increasingly recognized as a critical link between gut ecology and local bladder pathology. Its research value is growing, and it holds promise as a potential target for novel adjuvant therapeutic strategies.

### 3.5 Integrated mechanism: trans-organ signaling of the gut-bladder axis

**Figure 1:**
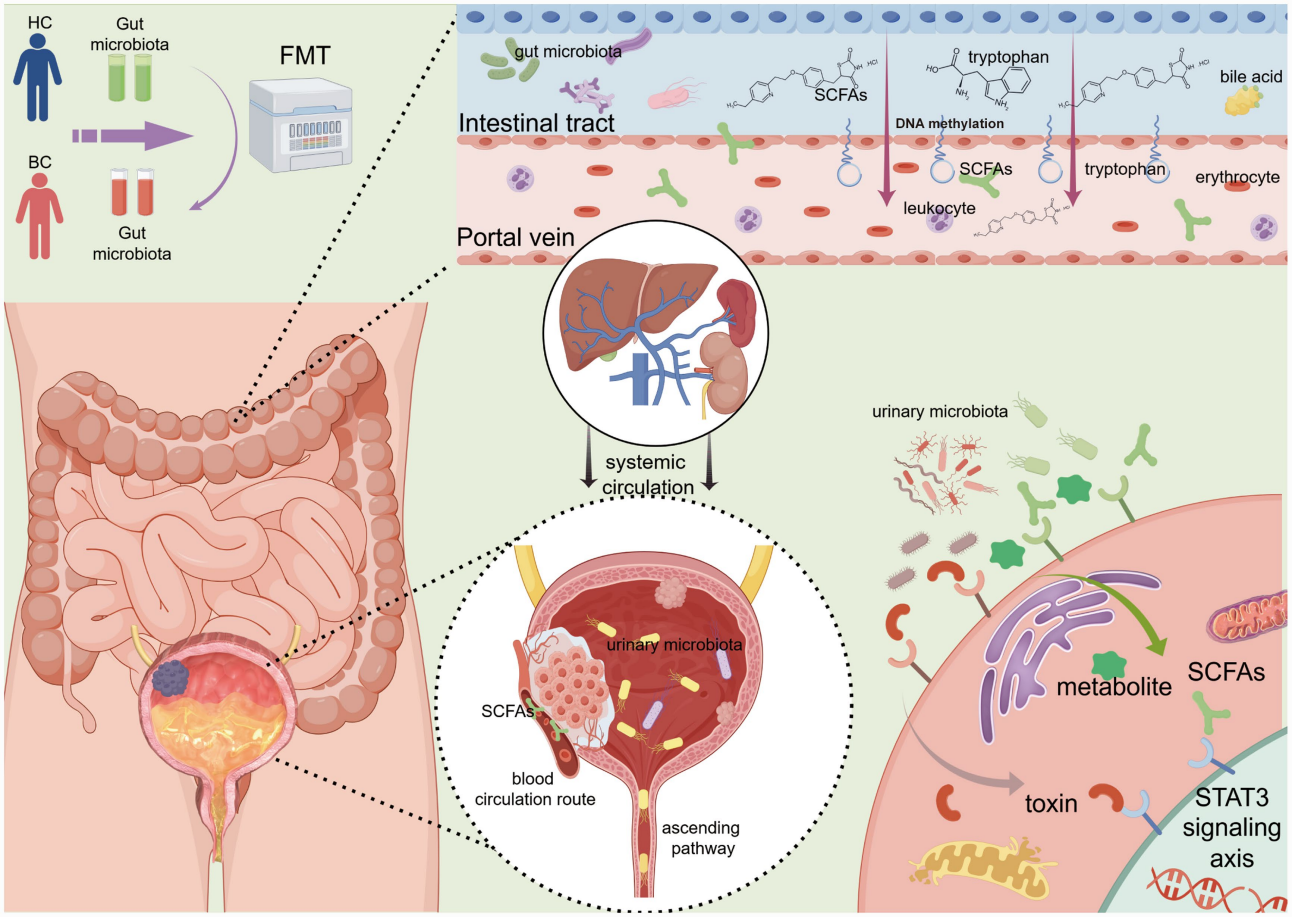
Potential mechanisms of FMT in bladder cancer treatment. This schematic illustrates the proposed multi-step signaling pathway. In the gut, a remodeled microbiota produces key metabolites (e.g., short-chain fatty acids, tryptophan derivatives, and bile acids). These metabolites are absorbed through the intestinal epithelium, enter the portal circulation, may undergo hepatic modification, and then disseminate systemically. Upon reaching the bladder, these gut-derived signals may act directly on tumor and stromal cells and/or indirectly modulate the local urinary microbiome. Collectively, these pathways converge to shape the bladder tumor microenvironment, thereby potentially influencing cancer progression and therapeutic response.

In summary, the potential mechanisms by which FMT influences bladder cancer through gut microbiota modulation may rely on a complete “gut-bladder axis” signaling pathway. The initiating step of this pathway is likely the production of key signaling molecules by gut microbiota, such as tryptophan derivatives, short-chain fatty acids, and bile acids. These molecules depend on an intact intestinal mucosal barrier for absorption into the bloodstream through the intestinal epithelium and subsequently enter the liver via the portal vein for potential metabolic transformation (Schiweck et al., 2022). Following this, they enter systemic circulation and may be further delivered to the bladder through the bloodstream (Yang et al., 2025). Within the bladder, these circulating metabolites may, on one hand, directly act on tumor cells. On the other hand, they may indirectly influence the composition and function of the urinary microbiota by altering urine physicochemical properties and the state of the bladder epithelium. This process could form a cascade-amplifying regulatory network, ultimately reshaping the bladder tumor microenvironment. The potential mechanisms of FMT are summarized in Figure 1.

## 4 Potential applications of FMT in modulating bladder cancer treatment

**Figure 2:**
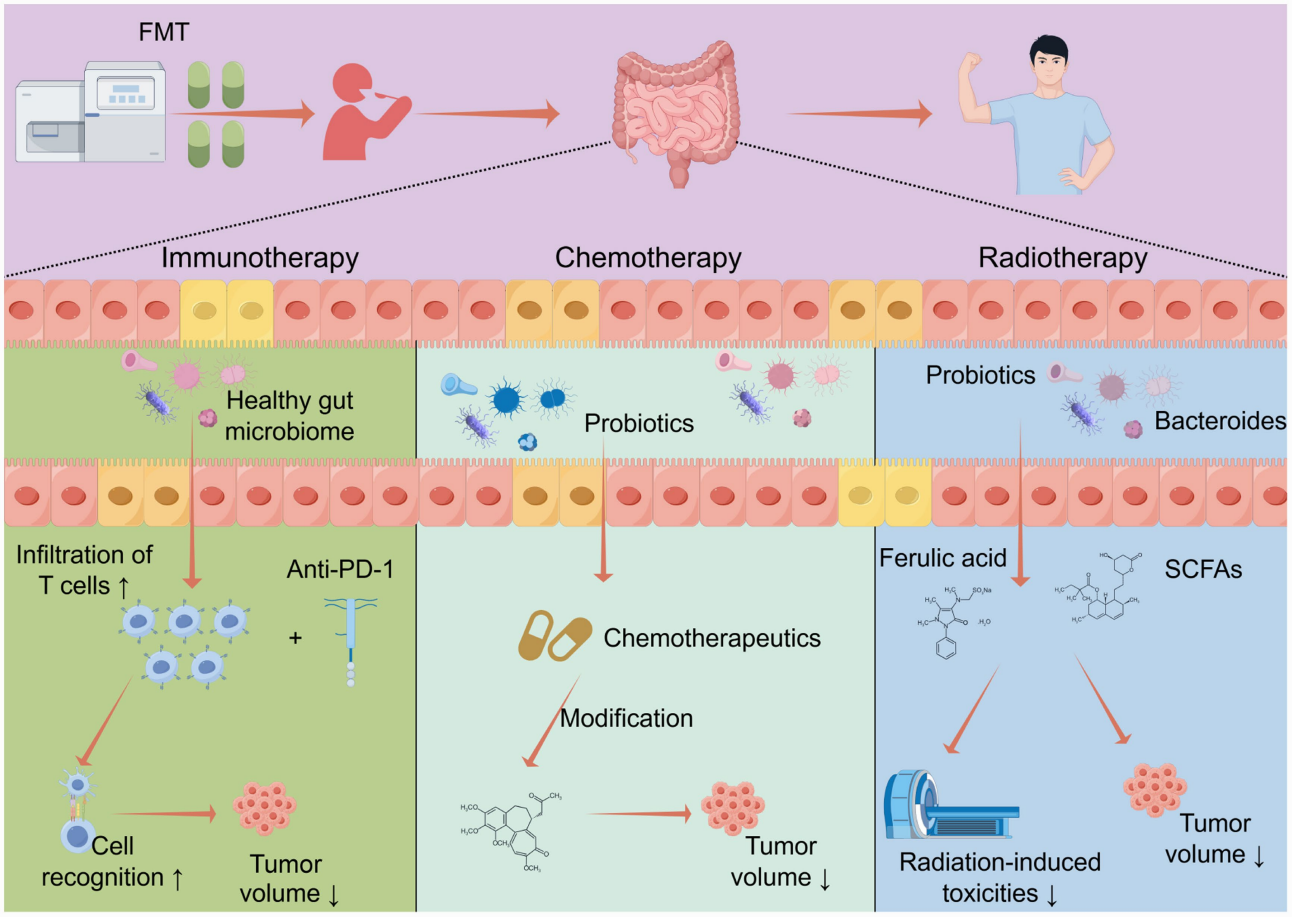
Potential applications of FMT in modulating bladder cancer treatment.

Three key application scenarios of FMT as an adjunctive therapy in bladder cancer are illustrated in Figure 2.

### 4.1 Immunomodulation: FMT as a potent immune modulator for bladder cancer

FMT is an emerging strategy to remodel the tumor immune microenvironment and has shown significant potential in enhancing the efficacy of tumor immunotherapy. The effectiveness of immune checkpoint inhibitors (ICIs) depends on an “immune-inflamed” tumor microenvironment. However, most bladder cancers present as either an “immune-desert” or “immune-excluded” phenotype, lacking infiltration of cytotoxic T cells, which represents a core bottleneck underlying primary resistance to treatment (Bellone and Elia, 2017; Kandalaft et al., 2022; Yu et al., 2023). This bottleneck may be overcome through the systemic immunomodulatory functions of gut microbiota. As a key “remote regulator,” gut microbiota can systemically shape antitumor immune responses by modulating macrophages, T/B cells, and cytokines (Ma et al., 2025). Hence, the value of FMT becomes evident: by transplanting a complete healthy microbial community, it can rapidly remodel the patient’s gut ecosystem, thereby precisely introducing and enriching key bacterial taxa with immune-activating functions (Yang et al., 2024).

Emerging research evidence is progressively validating the feasibility of this approach. Furthermore, preclinical studies have demonstrated that FMT can effectively reverse the host’s state of immune inertia (Xu et al., 2025). For instance, a high abundance of *Blautia coccoides* can promote the infiltration of CD8^+^ T cells into tumor sites through its metabolites, thereby enhancing the efficacy of bladder cancer immunotherapy. More importantly, combining FMT from healthy donors or specific beneficial bacteria such as *Parabacteroides distasonis* with anti-PD-1 therapy has been shown to synergistically increase the accumulation of intratumoral CD4^+^ and CD8^+^ T cells and significantly suppress bladder tumor growth (Wang et al., 2024; Wang et al., 2024). Notably, antibiotic-induced gut dysbiosis can significantly compromise the efficacy of ICIs. A study revealed that in urothelial carcinoma patients receiving ICI therapy, antibiotic exposure was strongly associated with markedly shortened overall survival and progression-free survival (Febriyanto et al., 2024).

The potential of FMT to reverse immune dysregulation has also been validated in clinical practice. A case report demonstrated that a bladder cancer patient who developed severe colitis following ICI treatment achieved steroid-free complete remission within one week after receiving FMT. Post-FMT stool analysis confirmed successful engraftment of donor-derived Bacteroidetes and *Akkermansia* species (Wang et al., 2018). This observation aligns with preclinical findings, collectively suggesting that precise modulation of the gut microbiota via FMT may transform immune-unresponsive “cold” bladder tumors into “hot” tumors, thereby offering novel strategies for managing immune-related adverse events.

### 4.2 Chemosensitization: FMT as a synergistic strategy for bladder cancer chemotherapy

Beyond its applications in immunotherapy, FMT also demonstrates potential in modulating chemotherapy efficacy. Although cisplatin-based neoadjuvant chemotherapy (NAC) remains the standard treatment for MIBC and provides a 5–9% recurrence-free survival benefit, its overall response rate remains suboptimal (Rouprêt et al., 2011; Park et al., 2025). This challenge underscores the importance of understanding host factors that influence chemotherapy sensitivity, with the gut microbiota emerging as a key mediator of interindividual variability in treatment response (Alexander et al., 2017). FMT influences chemotherapy response through two primary mechanisms: systemic immunomodulation and direct microbial metabolism of drugs (Böhm et al., 2025). Evidence indicates that gut microbiota-encoded enzymatic systems can directly modify chemotherapeutic agents such as cisplatin, thereby altering their pharmacokinetics and therapeutic efficacy (Zimmermann et al., 2019; Li et al., 2024). Moreover, specific gut microbiota compositions are closely associated with chemotherapy responses. A multicenter prospective study confirmed that bladder cancer patients with a high baseline abundance of Bacteroides exhibited poorer responses to NAC (Bukavina et al., 2024). This suggests that FMT may counteract such negative predictive factors by establishing a “favorable” microbial community, thereby optimizing the baseline therapeutic profile.

On the other hand, chemotherapy itself could induce gut microbiota dysbiosis. For instance, treatment with gemcitabine (administered alone or in combination with cisplatin) has been shown to significantly reduce microbial diversity (Miyake et al., 2023). This treatment-induced secondary dysbiosis consequently creates a therapeutic window for FMT intervention. Preclinical studies provide proof-of-concept for this approach, demonstrating that probiotic supplementation can enhance the antitumor efficacy of gemcitabine against bladder cancer through modulation of the gut-immune axis (Miyake et al., 2023). This suggests that FMT, as a more comprehensive and potent microbial restructuring strategy, possesses greater pre-sensitization potential. Consequently, FMT offers a potential novel approach to pre-emptively shape the gut microbiome into a state that supports rather than hinders chemotherapy efficacy, thereby providing new directions for overcoming the current chemotherapeutic bottlenecks in bladder cancer treatment.

### 4.3 The dual role of FMT in radiotherapy: toxicity reduction and radiosensitization

Pelvic radiotherapy serves as an important alternative curative treatment for MIBC, particularly for patient ineligible for radical cystectomy. However, its widespread adoption is constrained by two major factors: intrinsic tumor radioresistance and radiation-induced damage to normal tissues, especially the intestinal tract (Efstathiou et al., 2009). This condition could lead to radiation-induced enteritis, triggering a range of clinical complications such as diarrhea, perforation, and obstruction, thereby significantly constraining both treatment implementation and patients’ quality of life (Tirado et al., 2021; Murthy et al., 2025). The core pathophysiology primarily involves radiation-induced disruption of the intestinal multiple protective barriers, encompassing the physical, chemical, and immune components (Xin et al., 2022).

Encouragingly, gut microbiota and their metabolites have been found to possess dual potential, offering both radiosensitizing and radioprotective properties (Xin et al., 2022; Xie et al., 2024). FMT, as a potent approach for microbiota remodeling, holds promise for delivering synergistic effects in this field. In terms of radiosensitization, specific microbial community structures can enhance tumor response to radiation (Eaton et al., 2022). For instance, preclinical studies have confirmed that high dietary fiber intervention significantly delays tumor growth in tumor-bearing mice, with this radiosensitizing effect being closely associated with increased abundance of Bacteroidetes and elevated levels of the metabolite isoferulic acid (Then et al., 2024). In terms of radioprotection, FMT facilitates the restoration of damaged intestinal barriers, mitigates oxidative stress, and modulates local immune responses through the introduction of healthy microbiota, thereby alleviating radiation-induced enteritis. Specifically, higher abundance of probiotics such as *Lactobacillus* and *Akkermansia* is associated with improved survival outcomes, whereas enrichment of harmful bacteria like *Fusobacterium* exacerbates radiation-induced damage (Xie et al., 2024; Yi et al., 2023). Notably, the restoration of SCFA levels has been identified as a pivotal mechanism through which FMT mitigates radiation-induced toxicities (Then et al., 2024).

Thus, FMT holds promise as a novel dual-effect adjuvant that, when combined with radiotherapy, could enhance tumoricidal efficacy against bladder cancer while providing systemic protection for normal tissues, thereby broadening the therapeutic window of radiation.

## 5 From promise to practice: challenges in the clinical translation of FMT for bladder cancer

While FMT has demonstrated potential in treating recurrent *Clostridium difficile* infection (rCDI) and certain malignancies, its application in the field of bladder cancer continues to face significant challenges. To date, no clinical studies have been reported on the adjunctive use of FMT for bladder cancer. This gap largely stems from the anatomical separation between the intestines and the bladder, leaving the specific mechanisms by which gut microbiota influence the initiation and progression of bladder cancer still unclear. Consequently, more foundational research evidence is urgently needed. Drawing on experiences with FMT in rCDI and some urological diseases (such as complicated urinary tract infections), we will briefly outline the potential challenges of applying FMT in the context of bladder cancer treatment.

First, efficacy prediction and patient stratification represent a central challenge. The indications for FMT remain unclear, and reliable predictive models for treatment outcomes are lacking (Yadegar et al., 2024). Bladder cancer itself exhibits high genomic heterogeneity and complex molecular subtypes, which directly contribute to diverse clinical outcomes and treatment responses (Lopez-Beltran et al., 2024; Thomsen et al., 2016). This implies that a “simple microbiota transplantation” may be difficult to uniformly reverse all types of pro-carcinogenic microenvironments. Each molecular subtype, disease stage, and baseline response status to adjuvant therapies (such as immunotherapy, chemotherapy, and radiotherapy) may be associated with distinct gut microbial ecosystem profiles (Wang et al., 2024; Bukavina et al., 2024). The future focus lies in developing bladder cancer-specific biomarkers capable of predicting FMT efficacy. This requires the integration of bladder cancer genomics, host immune status, baseline gut microbial metagenomics, and other relevant data to enable a “precision” intervention approach.

Secondly, the standardization of treatment protocols is particularly complex within the context of combination therapies for bladder cancer. Unlike the “direct ecological restoration” approach used for rCDI and complicated urinary tract infections, FMT may be more suitable as an adjuvant therapy when applied to bladder cancer. Its design must account for the characteristics of the combined treatments. For instance, when combined with immunotherapy, should donors enriched with known immune-enhancing bacterial strains (such as Akkermansia) be selected (Routy et al., 2018)? When combined with radiotherapy or chemotherapy, should microbiota capable of mitigating treatment-related toxicities (such as butyrate-producing bacteria) be screened for (Sánchez-Alcoholado et al., 2021)? Furthermore, interventions such as antibiotic use and preoperative bowel preparation for bladder surgery can disrupt the gut microbiota, thereby impacting the engraftment of FMT. Consequently, there may not be a fixed standard for the “optimal FMT protocol” in bladder cancer; instead, it requires dynamic optimization based on concomitant therapies.

Third, safety considerations must be elevated in bladder cancer patients who may be immunocompromised (Kaver et al., 1992). While donor screening has significantly reduced infection risks, the potential consequences of pathogen transmission or microbial dysbiosis are more severe in patients who often experience immunosuppression. Particularly for patients receiving ICIs, FMT may reverse immune tolerance and enhance therapeutic efficacy, but it could also potentially aggravate immune-related adverse events (Routy et al., 2023; Halsey et al., 2023). This distinctive immunomodulatory characteristic underscore unique safety priorities when applied to bladder cancer, setting it apart from applications in infectious diseases.

Finally, the regulatory and ethical dilemmas surrounding FMT are becoming increasingly prominent. Unlike its use for rCDI or complicated urinary tract infections, FMT as an adjuvant therapy for bladder cancer lacks long-term safety and efficacy data. How to adequately inform patients about the long-term risks of this procedure poses a significant ethical challenge. Simultaneously, the classification of FMT as a biologic product remains unsettled, necessitating the establishment of a more rational regulatory review framework (Yadegar et al., 2024).

In summary, the application of FMT in bladder cancer treatment essentially involves transplanting a complete, healthy, and complex ecological community into a highly heterogeneous pathological environment. This not only requires addressing the universal challenges of FMT but also demands careful consideration of the pathophysiological characteristics of bladder cancer. Furthermore, more well-designed studies are needed in the future to substantiate the scientific rationale and clinical efficacy of applying FMT in bladder cancer treatment.

## 6 Conclusions and future perspectives

This review systematically outlines the theoretical basis and potential mechanisms of FMT in the treatment of bladder cancer, while analyzing the unique challenges in its clinical translation. Despite anatomical separation, compelling evidence indicates that the “gut-bladder axis” serves as a crucial long-distance communication pathway. It mediates bladder cancer initiation and progression by mechanisms involving microbial metabolites, immune regulation, barrier function, epigenetic reprogramming and modulation of the urinary microbiota. This makes the remodeling of gut microbiota via FMT to influence bladder cancer progression a highly promising scientific hypothesis.

However, there remains a long journey from theoretical research to clinical practice. The current core challenge lies in the disconnect between, on one hand, preclinical studies revealing abundant mechanisms of action and significant therapeutic potential, and on the other hand, the continued absence of direct clinical evidence, coupled with multiple challenges such as efficacy heterogeneity, technical standardization, safety concerns, and regulatory and ethical issues. Future research should focus on resolving these challenges: first, well-designed early-phase clinical trials are needed to verify the safety and preliminary efficacy of FMT in bladder cancer patients; second, multi-omics data must be integrated to identify biomarkers capable of predicting FMT efficacy, enabling precise patient stratification; finally, there is an urgent need to establish standardized FMT protocols suitable for combination therapy scenarios in bladder cancer and to promote the timely evolution of regulatory frameworks.

In summary, FMT may introduce a fundamentally new perspective to the bladder cancer treatment. It could represent a shift beyond the conventional “direct tumor-killing” approach, potentially aiming to reconstruct the host’s gut microbial ecosystem and thereby indirectly suppress bladder tumors. Although this strategy remains hypothetical in the context of bladder cancer, current preclinical and mechanistic insights suggest it holds promise as a novel therapeutic avenue worthy of future clinical investigation.

## Glossary

FMT: Fecal microbiota transplantation
MIBC: Muscle-invasive bladder cancer
NMIBC: Non-muscle-invasive bladder cancer
IDO1: Indoleamine 2,3-dioxygenase 1
KMO: Kynurenine-3-monooxygenase
TPH1: Tryptophan hydroxylase 1
TDO: Tryptophan-2,3-dioxygenase
SCFAs: Short-chain fatty acids
BAs: Bile acids
UDCA: Ursodeoxycholic acid
ICIs: Immune checkpoint inhibitors
NAC: Neoadjuvant chemotherapy
rCDI: Recurrent *Clostridium difficile* infection
MR: Mendelian randomization
IAA: Indole-3-acetic acid
HDAC: Histone deacetylase
FXR: Farnesoid X receptor
RXR: Retinoid X receptor
BSH: Bile salt hydrolase
CTGF: Cellular communication network factor 2
